# Protein Networks as Logic Functions in Development and Cancer

**DOI:** 10.1371/journal.pcbi.1002180

**Published:** 2011-09-29

**Authors:** Janusz Dutkowski, Trey Ideker

**Affiliations:** 1 Departments of Medicine and Bioengineering, University of California San Diego, La Jolla, California, United States of America; 2 Institute for Genomic Medicine, University of California San Diego, La Jolla, California, United States of America; Stanford University, United States of America

## Abstract

Many biological and clinical outcomes are based not on single proteins, but on modules of proteins embedded in protein networks. A fundamental question is how the proteins within each module contribute to the overall module activity. Here, we study the modules underlying three representative biological programs related to tissue development, breast cancer metastasis, or progression of brain cancer, respectively. For each case we apply a new method, called Network-Guided Forests, to identify predictive modules together with logic functions which tie the activity of each module to the activity of its component genes. The resulting modules implement a diverse repertoire of decision logic which cannot be captured using the simple approximations suggested in previous work such as gene summation or subtraction. We show that in cancer, certain combinations of oncogenes and tumor suppressors exert competing forces on the system, suggesting that medical genetics should move beyond cataloguing individual cancer genes to cataloguing their combinatorial logic.

## Author Summary

Biological outcomes are often determined by modules of proteins working in combination. In classic biological studies, these modules have been shown to encode a diverse repertoire of logic functions which provide the means to express complex regulatory programs using a limited number of proteins. Here, we integrate gene expression profiles and physical protein interaction maps to provide a systematic and global view of combinatorial network modules underlying representative developmental and cancer programs. We develop a new method that associates decision trees with concise network regions to identify network decision modules predictive of biological or clinical outcome. The resulting network signatures prove robust across different sample cohorts and capture causal mechanisms of development or disease. Furthermore, we find that the most predictive network decision functions rely on both coherent and opposing gene activities. Notably, in cancer progression the predictive gene associations often map to physical interactions between known oncogenes and tumor suppressors, where the combined activity of these genes determines disease outcome.

## Introduction

Biological complexity, it is thought, is not a simple function of the number of genes in a genome. It likely stems from a variety of factors, including the number of protein states and, as importantly, the number of combinations in which proteins assemble into functional modules [1], [2]. In development, it is largely combinatorial modules of transcription factors that give rise to the diversity of tissues [3]. Protein combinations are equally instrumental in the pathogenesis of human disease, for instance the inappropriate fusion of Bcr and Abl that leads to chronic myelogenous leukemia [4] or the abnormal interactions acquired by the huntington protein in Huntington's Disease [5].

An intriguing question is how the states of single proteins jointly determine the higher level states of protein modules. In classic biological studies, protein modules have been shown to encode basic logic functions such as AND, OR and NOT which are further combined within larger modules to code for complex programs [6]. A canonical example is the pigment cell module in sea urchin embryos [7]. There, the SuH/Groucho repressor complex forms in the absence of N^ic^ which, in turn, is determined by the lack of Delta signaling. Once Delta signaling is received, the SuH/Groucho repressor complex is displaced by the SuH/N^ic^ activator complex, which activates the GCM gene to induce pigment cell specification. In this case, the module activity can be summarized using basic AND and NOT functions:

IF Groucho AND SuH AND NOT N^ic^  THEN NOT GCM (NOT Pigment Cell)

IF N^ic^ AND SuH      THEN GCM (Pigment Cell)

Another example of network-encoded logic is the BAF chromatin remodeling complex [8]. The stem-cell specific version of the complex (esBAF) is characterized by presence of BRG1 but not BRM, and BAF155 but not BAF170 [9]. The neuron-progenitor version (npBAF) contains both BAF155 and BAF170 and also incorporates BRM and BAF60C while excluding BAF60B [10]. Pathological forms of BAF have also been characterized. For example the core subunit of the complex, SNF5, is inactive in malignant rhabdoid tumors, a highly aggressive cancer of early childhood [11].

**Figure 1:**
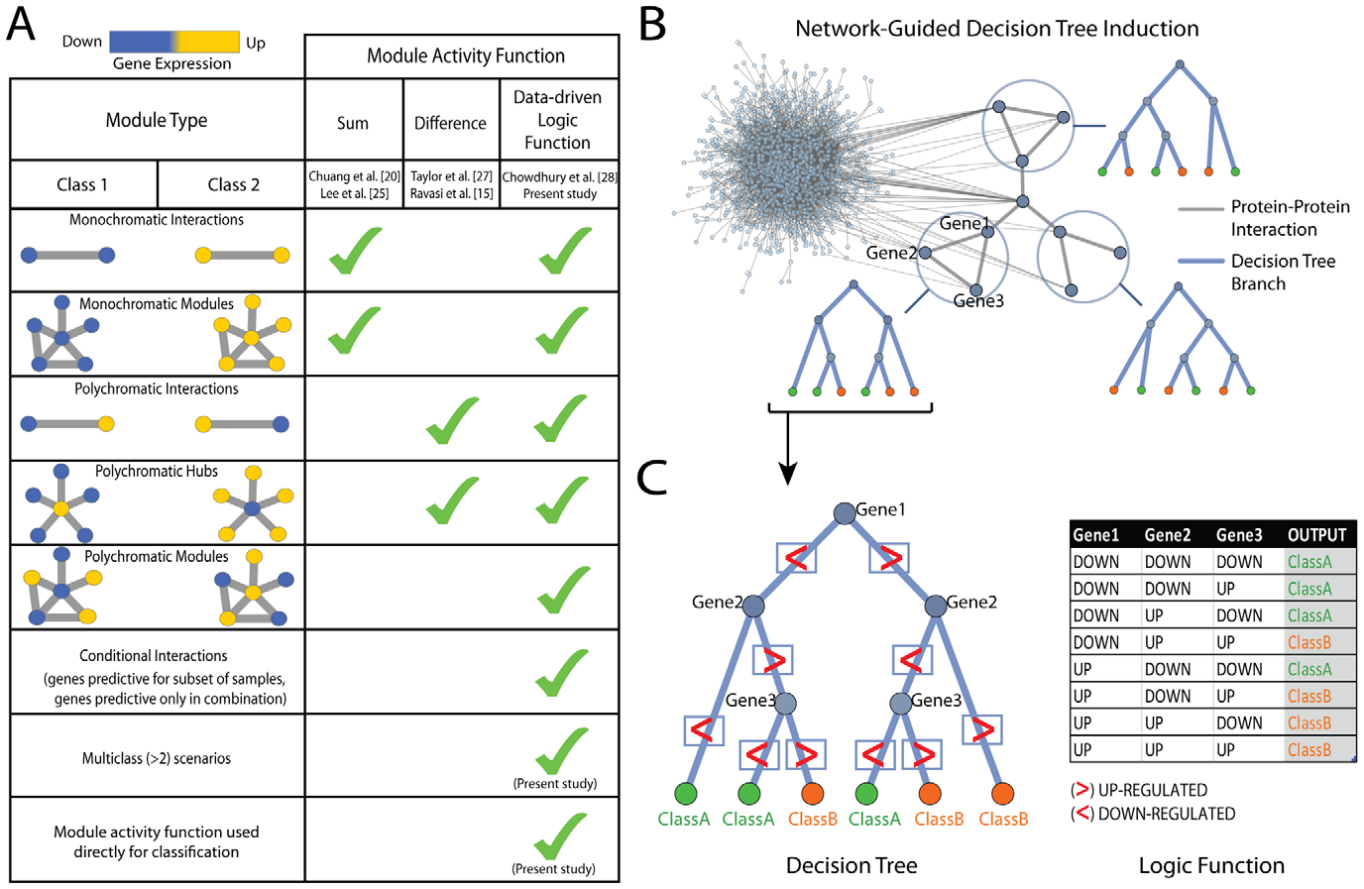
Method overview. (**A**) Representative module activity functions used by previous methods are compared to logic functions considered in this study. Logic functions capture a wide range of differential activities that are not captured by any single function. Our method uses logic functions directly in the classification process and extends to classification scenarios with more than two classes. (**B**) Network-guided search for decision trees associated with network modules. Each decision tree maps to a connected subnetwork. (**C**) Decision tree and the corresponding logic function represented as a truth table. The decision tree assigns each sample to a class by performing a series of tests where each test determines whether the expression of a selected gene is higher (>) or lower (<) than a threshold value. The gene is interpreted as being up-regulated if its expression is above the threshold. Otherwise the gene is down-regulated. Each path from root to leaf in the tree defines a single decision rule which maps to a different row in the truth table. Decision trees are typically not grown to the full extent and thus not all genes must be tested along each path if a subset of the genes is sufficient to determine the output.

Given the importance of protein modules and their outputs, a major activity within the field of Systems Biology has been to identify such modules systematically through analysis of global data sets [12]–[16]. Many computational methods have been developed to integrate a panel of gene expression profiles with protein-protein interaction maps or pathway databases, with the goal of associating modules with a biological or clinical outcome [17]–[30]. Among these, several approaches have investigated how protein modules can be used to classify samples. In these methods, each module defines a set of interacting proteins whose expression levels are combined to determine the module activity, which in turn is used to predict the phenotypic class of the sample. However, with one recent exception [28] these methods have assumed that the activity of every module of interest is homogenous and follows a single general function, such as the sum of gene expression levels in a module [20], [25] or the difference in expression levels across interacting genes in a module [15], [27] (**Figure 1A**). While these simple functions (as well as more advanced frameworks [22], [24], [29]) can identify coherently expressed or perturbed modules, they do not provide the rich logical framework known to occur in biological systems.

Here, we develop a novel method called Network-Guided Forests (NGF) to learn the network modules whose logic specifies key biological and clinical outcomes. NGF integrates key ideas from Random Forests (RF) [31] with biological constraints induced by a protein-protein interaction network— the first use of protein networks in ensemble learning [32]. Rather than relying on a general measure of module activity, NGF fits specific logic functions to each module directly from data. In contrast to Chowdhury *et al.* [28] who learned network state functions to select informative gene sets that were further used to train a neural network model, the functions identified here are used directly in the classification process. NGF can also readily be applied to continuous gene expression measurements and problems with more than two classes. Using NGF, we explore the functions used in diverse biological programs related to tissue differentiation, breast cancer metastasis, or mesenchymal transformation of brain tumors. For each case a set of network modules is identified which captures known causal mechanisms of development or disease and – in contrast to classical Random Forests – provides robust biomarkers across different sample cohorts. The modules implement diverse logic functions using both coherent and opposing gene activities, in which the module output depends on expression increases for some genes and concomitant decreases for others. Notably in cancer progression, the most predictive decision functions can often be linked to interactions between known oncogenes and tumor suppressors, such that the combined activity of both types of genes determines the disease outcome.

## Results

### Overview of NGF approach and data

The NGF framework learns a set of decision trees (the “forest”) in which each tree maps to a connected component of the protein-protein interaction network (**Figure 1B**). The decision tree specifies a function that determines the output of the network component based on the activity of its genes. In turn, the collection of all tree outputs is used to predict the cell type or disease state of the biological sample (the “class”). When binary gene activities and two-class decision problems are considered, decision trees map directly to Boolean logic functions [33] (**Figures 1C** **, S1**). In general, however, decision trees can be readily applied to continuous gene activity values and multi-class scenarios [**34**].

To build a decision tree, NGF selects an initial gene to partition the samples by high versus low gene expression and it scores how well this partition separates the classes. Samples for which the expression of the selected gene is high are placed in the right subtree while those for which the expression is low are placed in the left subtree. NGF then conducts a network-guided search which progressively adds new genes to the tree to improve its discrimination between classes, with new genes chosen from the network neighborhood of genes already in the tree (**Figure 1B**; Materials and Methods). Many trees are built, starting from many different initial genes, to define the forest.

By construction, decision trees include genes that influence a phenotypic outcome both individually and through multi-way interactions with other genes [35]. As in the standard Random Forests algorithm, NGF uses a permutation-based procedure to assess the importance of each gene on the classification accuracy of the forest (Materials and Methods). Motivated by [36], we also assess the importance of pairs of genes in a tree — in our study these pairs are constrained by the network neighborhood. Genes and gene pairs with significantly high importance scores are placed into clusters that capture similar patterns of presence/absence across the forest of decision trees. Each cluster aggregates genes that fall into the same network region and, in combination, have predictive power over the sample class. Hence these clusters are termed “consensus decision modules”.

**Figure 2:**
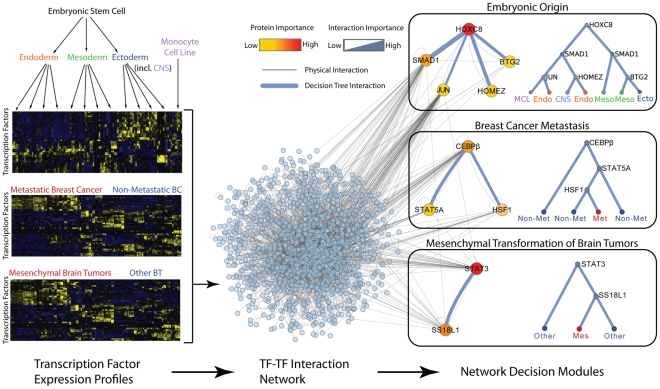
Network decision modules underlying embryonic origin, breast cancer metastasis and mesenchymal transformation of brain tumors. Expression profiles for each of the three case studies are combined with a network of protein-protein interactions among human transcription factors. Network-guided forests are used to identify key network modules that are most important for correct sample classification (representative modules are shown for each study). Grey edges indicate physical protein-protein interactions, blue edges indicate protein combinations that often co-occur in the same decision trees and are most important for classification (as indicated by the permutation test). Node color indicates protein importance whereas edge width indicates the importance of a protein combination. Each module is assigned a decision tree that specifies the output of the module based on the activity of its genes (see also Figure S1).

To apply this framework to study the logic of biological decisions, we obtained mRNA expression data from three diverse studies related to (1) Development of germ layers, (2) Breast cancer metastasis, or (3) Progression of glioma, respectively (Materials and Methods). While these studies collectively span a wide range of human biology, each makes use of mRNA expression profiles to discriminate between classes of development (study 1) or disease (studies 2 and 3). To provide a complementary protein network, we downloaded a set of 5227 physical interactions measured among pairs of human transcription factors, many of which have been recently reported using the mammalian two hybrid system [15]. NGF was used to combine this protein network with each expression data set to derive a forest of decision trees and corresponding network decision modules for each study (**Figure 2**). To allow comparison to other module-finding approaches, we also obtained a network of 57,228 human protein-protein interactions as used previously in [20], [24]. Further information about each expression and network data set is provided below and in **Table S1**.

### Network modules reveal causal mechanisms of development and are robust

Tissue differentiation is largely governed by combinatorial interactions among transcription factors [1]. To identify protein modules involved in tissue development, we applied NGF to qRT-PCR expression profiles collected for 34 human tissues (Ravasi et al. dataset [15]) classified according to their embryonic origin: endoderm, mesoderm, non-neural ectoderm, central nervous system (CNS) or cell lines (**Figure 2**). NGF integrated these data with the transcription factor protein interaction network (**Table S1**) to reveal a set of 16 consensus decision modules, each containing genes frequently used in combination to predict tissue origin (**Figures 2** **,** **3A**). Among these modules, we recognized a number of well-established regulatory complexes with known decisive roles in development (**Table 1**). For instance, the single most predictive interaction identified was between HOXC8 and SMAD1, a transcriptional heterodimer that is known to induce osteoblast differentiation [37]. Also consistent with the logic identified by NGF (**Figure 2**), HOXC8 is highly expressed in ectoderm and mesoderm during mouse early embryogenesis [38].

**Figure 3:**
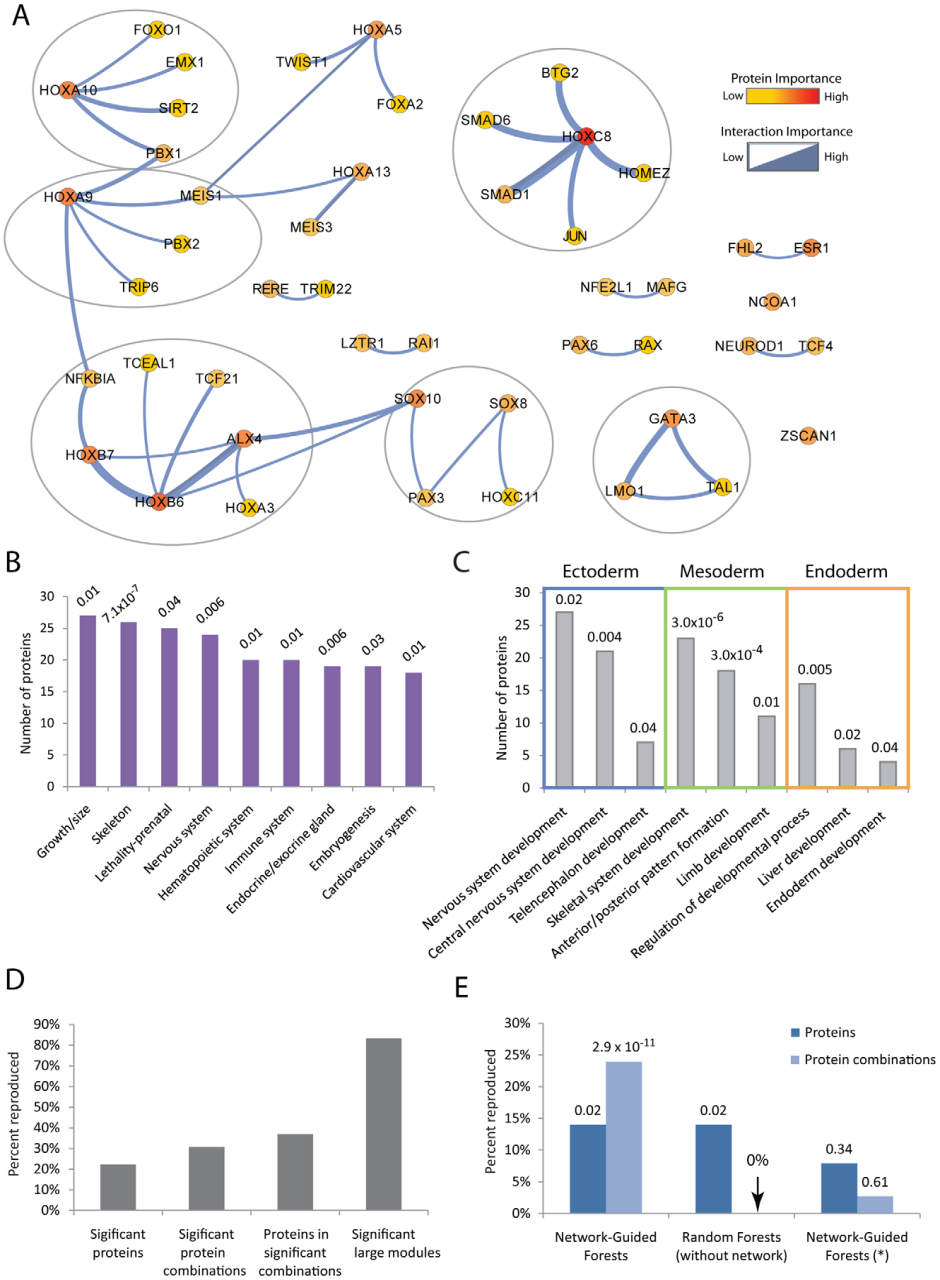
Network modules capture causal developmental factors and are reproducible. (**A**) Consensus network modules underlying tissue origin (modules of size greater than 2 are encircled). Gene pairs that often co-occur in the same decision trees and are most important for classification are shown in blue. Node color indicates protein importance whereas edge width indicates the importance of a protein combination. (**B**) Enrichment for developmentally-related phenotype categories in the MGI database (FDR is reported above each bar). (**C**) Enrichment of germ-layer specific genes identified by NGF based on the Gene Ontology (FDR is reported above each bar). (**D**) Percentage of genes, interactions, and modules that were reproduced based on an independent dataset. (**E**) Percent of reproduced single genes and gene combinations (Fisher's Exact Test P-values are reported). NGF* indicates the result for NGF applied to networks with perturbed expression measurements.

**Table 1:** Network modules corresponding to known regulatory complexes in development.

Module	Known role/tissue specificity	References
GATA3-LMO1-TAL1	Activates the transcription of RALDH2 in T-cell Acute Lymphoblastic Leukemia	[64]
HOX-PBX-MEIS-SMAD	Potential for higher order complexes that modulate tissue activity	[54]
HOXA5-TWIST1	HOXA5 partially restores inhibitory effects of Twist on p53 target genes in breast cancer cells	[65]
HOXA9-PBX1-MEIS1	Regulates CYBB transcription in myeloid differentiation	[66]
HOXA10-SIRT2	Promotes histone deacetylation; represses gene transcription	[67]
HOXB7-NFKBIA	NF-κB and IκB-α increase transactivation by HOXB7	[68]
HOXC8-SMAD1	Promotes osteoblast differentiation	[37]
HOXC8-SMAD6	Hoxc8 represses BMP-induced expression of Smad6	[69]
PAX3-SOX10	Mediates activation of c-RET enhancer in neural crest precursor cells	[70]

A systematic functional analysis of the modules (Materials and Methods) indicated that they were highly enriched for genes whose perturbation is linked to prenatal lethality or improper organ development in mammals (**Figure 3B**), as reported in the Mouse Genome Informatics (MGI) database [39] — an established source of functional associations for both mouse genes and their human orthologs [13]. Gene Ontology analysis [40] indicated that the network was significantly enriched for pattern-specification homeobox genes (19/48 genes) and other developmentally important gene categories, for example embryonic morphogenesis and skeletal system development (**Figure S2**). Furthermore, we found that the genes used by NGF to identify a particular tissue origin (endoderm, mesoderm, ectoderm) were generally implicated in developmental processes specific for that type of tissue (**Figure 3C** and **Text S1**).

To examine the robustness of these decision modules, we investigated whether they could be reproduced from random subsets of the input gene expression profiles, as well as from an independent set of profiles. We found that the protein combinations co-occurring within the same module were highly reproducible across subsets of expression profiles, much more so than the protein combinations identified by the standard Random Forest algorithm (**Figure S3**). Further, NGF was used to analyze a large expression profiling study by Muller et al. [13] consisting of 153 types of multipotent stem cells, where each cell type is attributed to the mesoderm, endoderm or ectoderm. We analyzed the single proteins and protein pairs identified as being significantly predictive in the previous dataset (Ravasi et al.; **Figure 3A** **)** and compared them to the same number of top scoring proteins and protein pairs identified in the dataset from Muller et al. While only two of ten significant proteins (20%) were identified in common based on single feature analysis, we found that 14 of 38 proteins (37%) were reproduced based on importance scores for pairs of genes (**Figures 3D** **, S4**). Among non-trivial decision modules (i.e., those with three or more proteins), five out of six (83%) were recovered in both studies (**Figures 3D** **, S4**). In comparison, the standard Random Forest algorithm, which did not use the network, was not able to identify any reproducible gene combinations (**Figure 3E** **; Text S1**). Moreover, randomized runs of NGF (in which the assignment of expression profiles to network nodes was permuted) identified only 8% of the same genes and 3% of the same gene-gene combinations (**Figure 3E**). Taken together, these results indicate that the tissue-specific network expression pattern identified by NGF is both biologically relevant and robust across sample cohorts.

### Informative and robust models of breast cancer and glioma progression

**Figure 4:**
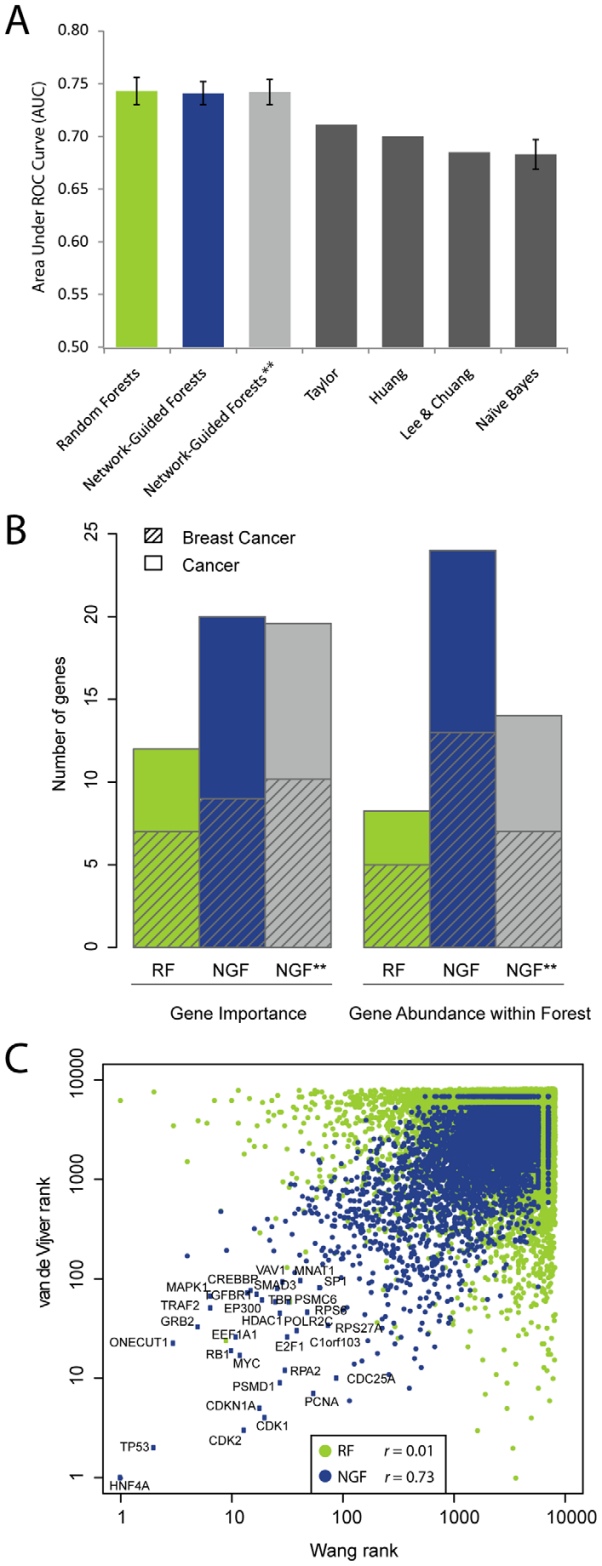
Classification performance and validation of markers of breast cancer metastasis. (**A**) Average area under the ROC curve for NGF, RF, NGF applied to permuted networks (NGF**), and Naïve Bayes, compared to reported scores for representative previous methods (error bars denote standard deviation estimated over 100 runs). (**B**) General cancer and breast cancer associated genes identified among the 100 top-scoring genes or 100 most abundant genes in the forest created using RF or NGF. using the real network or networks with permuted edges (average over 100 permutations is shown). (**C**) Genes ranked by their importance for classification in two independent breast cancer patient cohorts (y vs. x axis). Network-Guided Forest, blue points; regular Random Forest, green points.

While normal developmental programs are tightly regulated, pathological states including cancer can reflect regulatory programs gone awry. To investigate how well NGF can predict cancer progression and identify robust biomarkers, we selected a cohort of 295 nonfamilial breast cancer patients (van de Vijver dataset [41]), for 78 of whom metastasis has been detected during a follow-up visit within five years after surgery. The accuracy of NGF and other algorithms in classifying metastatic vs. non-metastatic samples was assessed using a five-fold cross validation scheme repeated 100 times. The average area under the ROC curve (AUC) for Network-Guided Forests was 0.74 (**Figures 4A** **, S5A**), which was better by 3–6% than previously reported results for a variety of standard and network/pathway-based classification methods [**24]**, [25], [27****].

Interestingly, the performance of NGF was on par with regular Random Forests (non-network-based), as well as with NGF applied to randomized networks in which the edges were permuted while maintaining the original degree distribution (NGF**; **Figures 4A** **, S5A**). Thus, it appears that the decision tree framework used by all three methods is able to find predictive feature sets regardless of the restriction imposed by the protein-protein interaction network. However, in contrast to Random Forests we found that NGF identified many more genes with known roles in breast cancer or cancer in general (**Figure 4B**). Closer inspection showed that known cancer genes are often not among the most differentially expressed, but are predictive in combination with their network neighbors so that they appear among the most abundant genes in the forest (**Figure 4B**). In contrast, permuted networks identified far fewer cancer genes among the most abundant features, indicating that the network neighborhood provides crucial information which guides NGF to the biology of disease.

To study the robustness of markers identified by NGF, we compared the most abundant features from the van de Vijver dataset to those found in an independent study of 106 metastatic and 180 non-metastatic breast cancer samples described by Wang et al. [42]. The correlation of the resulting gene rankings based on their occurrences in the forest was 0.73 for NGF versus 0.01 for the regular Random Forest algorithm. Altogether, 31 genes were shared among the 100 most abundant genes from the two datasets, compared to 2 common genes identified by Random Forests (**Figure 4C**). Thus, the regularization imposed by the network serves to focus the training process on true cancer susceptibility genes, which are observed reproducibly across data sets.

These general findings were also observed in a different process related to cancer progression: mesenchymal transformation of brain tissue. Mesenchymal transformation has been associated with exceedingly aggressive forms of high-grade gliomas (HGGs) – the most common type of brain tumor in humans. To study network activity patterns leading to the mesenchymal phenotype, we trained the NGF framework on expression profiles of 76 HGG samples previously assigned to one of three groups: proneural, proliferative or mesenchymal [43]. Proneural and proliferative samples were grouped together as “non-mesenchymal” and treated as a control group for detecting the mesenchymal network signature. As with breast cancer, we found that NGF outperformed the benchmark classifier Naïve Bayes in terms of classification accuracy and performed as well as the standard Random Forest algorithm (**Figures S5B, S6A**). Furthermore, NGF identified more cancer susceptibility genes among the top ranked features (**Figures S6B**).

### Logic functions embedded in protein networks

**Figure 5:**
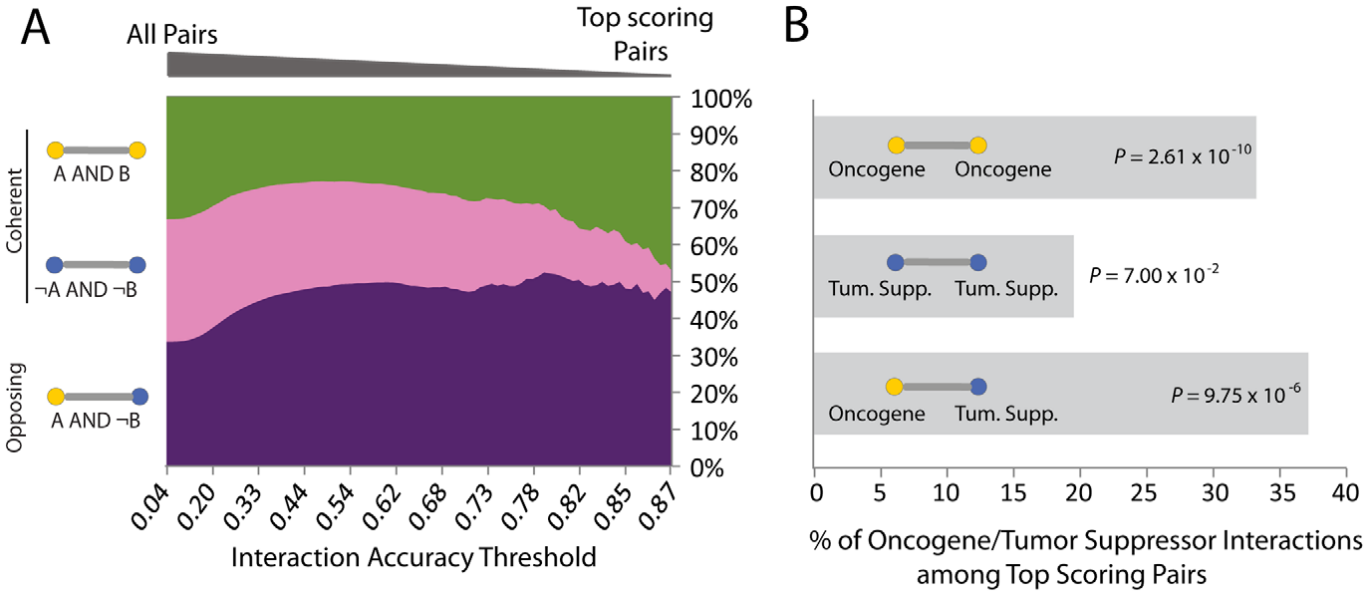
Network functions underlying cancer progression. (**A**) The decision trees for mesenchymal transformation are dissected by assigning their gene pairs to one of three functional categories based on the sign of gene expression in predicting the more aggressive phenotype. The percentage of gene pairs assigned to each of the three functional categories is shown as a function of the score threshold used for selecting gene pairs. Accuracy is calculated as the average Laplace score (**Text S1**) over all trees in the forest. (**B**) Enrichment for interactions between oncogenes, between tumor suppressors and between an oncogene and a tumor suppressor among functional categories identified using NGF. Percent of such interactions among top scoring pairs in each functional category is reported along with the Fisher's Exact Test P-value of enrichment.

We next wished to determine whether there were particular network decision functions that were common across biological data sets or, alternatively, which functions were distinct. For this purpose, protein interactions in the decision trees were functionally categorized according to the sign of their proteins in classifying a given phenotype (**Figure 5A** **; Text S1**). The three functional combinations were: “A AND B”, “NOT A AND NOT B” and “A AND NOT B”. We asked which of these functions can best separate the samples into class-homogeneous groups and which types of functions are preferred.

Indeed, we found that particular functions were overrepresented among the most predictive gene combinations and that these functions differed across the different biological processes investigated (**Figure S7**). Interestingly, across all cancer datasets, decision functions used to predict the more aggressive phenotype were more likely to be associated with “A AND NOT B” logic than other functions (**Figures 5A** **, S7**). Such opposing gene combinations were instrumental in many decision modules identified by NGF. For instance, in breast cancer a highly predictive consensus decision module was identified among C/EBPβ, STAT5A, and HSF1 (**Figure 2**) – three genes whose activity has been shown to directly influence cancer progression [44]–[46]. The unfavorable metastatic phenotype is associated with high levels of C/EBPβ and HSF1 and low levels of STAT5A (**Figures 2** **, S1A**). Consistent with this prediction, upregulation C/EBPβ can induce acquisition of an invasive phenotype [44], and expression of HSF1 is required for cellular transformation and tumorigenesis in HER2-positive breast tumors [46]. STAT5, on the other hand, has been shown to inhibit invasive characteristics of human breast cancer cells and is often lost during metastatic progression [45]. Similarly, for the brain tumor case study, NGF identified a key logic function which associates the mesenchymal phenotype with the upregulation of STAT3 and downregulation of SS18L1 (**Figures 2** **, S1B**). STAT3 is a known oncogene recently identified as a driver of mesenchymal transformation in brain tumors [**14**], while SS18L1 is a protein normally required for calcium-dependent dendritic growth and branching in cortical neurons [47].

Across all functional categories, we found that the top scoring decision functions identified in cancer were enriched for interactions between known cancer-related genes (P = 4.92×10^−4^ and P = 1.94×10^−3^ for the mesenchymal transformation of brain tumors [43] and breast cancer metastasis [42], respectively). Moreover, opposing functional combinations (“A AND NOT B”) predictive of the mesenchymal transformation were significantly enriched for interactions between products of oncogenes and tumor suppressors (**Figure 5B**). In turn, the coherent combinations “A AND B” or “NOT A AND NOT B” were enriched for known interactions between oncogenes or between tumor suppressor genes, respectively (**Figure 5B** **; Table S2**). These results support a model in which the aberrant cancer-related activity is caused by combinations of oncogenes and tumor suppressors co-occurring in the same pathways [48]-[50] and suggest that decision modules reported by NGF may be an excellent means to identify such combinations for further study (**Table S2)**.

## Discussion

Previous efforts to mine networks for differentially-expressed modules have assumed that module activity can be represented with a single functional form. This hypothesis is expressed in the scoring function that is applied to each module to assess its differential activity. However, our analysis of a representative sample of diseases and developmental programs indicates that the most effective decision functions are in fact not homogeneous, but involve a combination of coherent and opposing gene-gene interactions.

While the biological programs covered in this paper are certainly not a comprehensive survey of molecular decision-making, it is significant that both the developmental and cancer modules lead to similar conclusions. First, the network signatures identified by NGF are robust as evidenced by their support from multiple independent datasets. Of the development modules reported by NGF, 83% are reproduced across developmental datasets, in contrast to 0% reproduced by a network-free approach. In breast cancer, we observed a 73% correlation between the features selected for breast cancer, in contrast to 1% for a network-free approach.

Second, while the overall classification performance of NGF does not differ from regular Random Forests, network information does achieve sharp focus on genes and gene combinations that are close to the causes of development or disease. A known difficulty with classification using molecular profiles is that it is possible to construct many alternative classifiers all of which have equivalent performance but are based on very different sets of genes [51], [52]. This is due to the relatively small number of samples as well as the large number of genes that are correlated with outcome. Among the many alternative classifiers, some rely on genes that are close to the true disease mechanisms, while most rely on distantly associated genes. NGF constrains the selected gene features to fall into contiguous protein interaction subnetworks. These network-derived features are more reproducible and strongly enriched in the expected gene functions: Developmental modules are highly enriched for development, and cancer modules are highly enriched for known cancer susceptibility factors. Thus the prior knowledge of the protein interactions serves to filter the set of all possible classifiers [53] allowing NGF to identify those that are based on biologically relevant markers.

Finally, network analysis reveals how single factors form predictive combinations. In development, NGF identifies a concise network of HOX genes interacting with developmentally important cofactors, whose tissue-specific roles are just beginning to be illuminated [37], [54]. In cancer, combinations of interacting oncogenes and tumor suppressors are found such that their combined activity determines disease outcome. Beyond development and cancer, it is likely that for many biological programs, molecular interaction networks will provide a useful framework to guide computational approaches towards biologically-relevant and reproducible genetic logic.

## Materials and Methods

### Datasets

Detailed information on gene expression and protein-protein interaction datasets is provided in **Table S1**. Phenotypes associated with genetic perturbations in mouse (**Figure 3B**) were downloaded from the MGI database [55]. Cancer-associated genes (including breast and brain cancer genes) were from the Genetic Association Database [56] and were downloaded from DAVID [57]. Lists of tumor suppressors and oncogenes were downloaded from the Cancer Genes database [58].

### Network-Guided Forests

NGF is a network-based supervised learning algorithm that constructs an ensemble of decision trees which vote to determine the class of a sample. As in the standard Random Forests algorithm, each tree is constructed based on a bootstrap subset of samples drawn with replacement from the original training set. The individual trees are built using the recursive partitioning algorithm CART (Classification And Regression Trees) [59]. CART uses a measure of impurity called the Gini index to determine how well a gene and a corresponding expression threshold can differentiate samples with respect to their phenotypic class. The best such gene establishes the first split in the tree. Samples for which the expression value for the selected gene is lower than the threshold are assigned to the left child node in the tree and those with values higher than or equal to the threshold are put in the right child node. This process is iterated for each child node until the improvement in class separation (as measured by the Gini index) is lower than *ε* (here we use *ε* = 0.02 or *ε* = 0.01 for the global and transcription factor-specific network, respectively). In NGF, as in Random Forests, the search process applied by CART is randomized to allow for multiple concurrent trees to be built. First, each tree root is selected as the best gene among a random subset of size √*N*, where *N* is the number of all considered genes. Then, at each subsequent node in the tree, the best splitting gene is selected among a random candidate set. NGF selects the candidate set among network neighbors of genes already present in the tree. To promote the identification of dense subnetworks, the roots are required to have at least *k* network neighbors (here *k* = 5) and the candidate set of subsequent nodes is expanded iteratively, where each time the probability of selecting a given gene for the candidate set is proportional to the number of interactions it shares with genes already in the tree. NGF also requires that each gene appears at most once on each path from the root to the leaf of the tree. After the trees are constructed, the entire forest is used to determine the class of a new sample. For each tree, the sample is propagated down from the root of the tree and assigned to one of the leaves according to the series of splitting conditions along the path leading from root to leaf. The probability of a given class is determined based on the proportion of training samples that were initially assigned to this leaf. The average probability across all trees is computed and the value of this score is used to determine sample class. Different score thresholds can be used to trade-off specificity and sensitivity.

### Identifying network decision modules

Following [31], we use samples that were not selected to construct a given tree (so called “out-of-bag” samples) to estimate the misclassification error of the tree and determine feature importance. Specifically, we use each tree to classify the corresponding out-of-bag samples and report the percentage of samples misclassified. Next, for each gene in the tree, we measure the increase in the misclassification error resulting from permuting the expression measurements for this gene in the out-of-bag samples. The mean increase of this error over all trees determines the importance score of each gene (trees in which a gene was not used are counted and contribute 0 to the mean). An analogous approach is used to determine the importance scores for pairs of genes. For this we calculate the mean increase in tree misclassification error caused by permuting expression values of any two genes which are used by a particular tree (see [35], [36], [60] for related techniques applied for standard decision tree ensembles). To construct network decision modules, NGF outputs the top scoring genes and gene pairs which have a False Discovery Rate (FDR) < 0.05, where the null distribution is estimated by executing NGF 100 times on data with permuted class labels. The stability of this procedure increases with the number of trees in the forest. For datasets used here, we found that the method produces robust results provided that the forest contains > 20,000 trees. For gene pairs, we additionally check that the mean increase in the misclassification error for the pair is significantly greater than for any single gene in that pair in trees where both genes are present (FDR<0.05). Genes with significant importance scores either independently or in combination with other genes are clustered based on how often they co-appear in the same decision trees. To this end we apply the affinity propagation algorithm [61] which is implemented as a plugin for Cytoscape [62], [63].

### Functional enrichment analysis

Gene Ontology enrichment analysis was performed using DAVID [57]. MGI phenotype enrichment and enrichment for cancer genes was calculated using Fisher's Exact Test implemented in R (http://www.R-project.org). All enrichments were calculated with respect to the background of all genes present in the input protein-protein interaction network used in each study.

## Supporting Information

Figure S1 **Modules, decision trees and logic functions.** The logic functions behind key modules for breast cancer metastasis (**A**) or brain tumors (**B**) are represented using decision trees and truth tables. In each case the gene is interpreted as being up-regulated if its expression is above the threshold. Otherwise the gene is down-regulated. Each path from root to leaf in the tree maps to a different row in the truth table. Decision trees are typically not grown to the full extent and thus not all genes must be tested along each path if a subset of the genes is sufficient to determine the output.
(TIF)

Figure S2 **Gene Ontology enrichment analysis.** Genes in the network identified by NGF (**Figure 3A**) are enriched for important developmental processes catalogued in the Gene Ontology. FDR is indicated above each bar.
(TIF)

Figure S3 **Robustness of NGF results in cross validation runs.** The average percentage of the top 50 proteins and top 50 protein pairs identified for the developmental case study (**A**), the breast cancer metastasis case study (**B**) or the brain tumor case study (**C**) that were reproduced on datasets with 10% of the data held-out. Error bars indicate standard deviations estimated over 100 runs.
(TIF)

Figure S4 **Overlap between NGF results based on Ravasi and Muller datasets. (A)** Network modules identified using NGF based on the Ravasi dataset were limited to genes available also in the Muller dataset. Large modules (3 or more proteins) are encircled. **(B)** Overlapping genes and interactions identified based on the Muller dataset. Conserved large modules for which at least one interaction is retained in the result based on the Muller dataset are encircled.
(TIF)

Figure S5 **ROC analysis.** Representative ROC curves for NGF, RF and NGF applied to networks with permuted edges (NGF**) for classification of breast cancer metastasis (**A**) and brain tumors (**B**). The average probability of a class computed across all trees in the forest is used as a parameter to trade off sensitivity and specificity.
(TIF)

Figure S6 **Classification performance and validation of network markers of mesenchymal transformation. (A)** Average area under the ROC curve for NGF, RF, NGF applied to networks with permuted edges (NGF**), and Naïve Bayes (error bars denote standard deviation estimated over 100 runs). **(B)** Cancer and brain cancer associated genes identified among 100 top-scoring genes or 100 most abundant genes in the forest created using RF or NGF using the real network or networks with permuted edges (NGF**, average over 100 permutations is shown).
(TIF)

Figure S7 **Network functions underlying development and cancer progression.** For each study, the percentage of gene pairs assigned to each of the three functional categories is shown as a function of the score threshold used for selecting gene pairs. Accuracy is calculated as the average Laplace score over all trees in the forest (**Text S1**).
(TIF)

Table S1 Protein-protein interaction networks and transcriptional profiles used in this study.
(DOC)

Table S2 Predictive interactions between known oncogenes and tumor suppressors identified among top-scoring gene pairs from the NGF analysis.
(XLS)

Text S1 **Supplementary methods.**
(DOC)
