# Supplementary figures and images for: Protein Networks as Logic Functions in Development and Cancer
Source: PLoS Comput Biol. 2011 Sep 29;7(9):e1002180. doi: 10.1371/journal.pcbi.1002180 (PMC3182870; doi:10.1371/journal.pcbi.1002180)

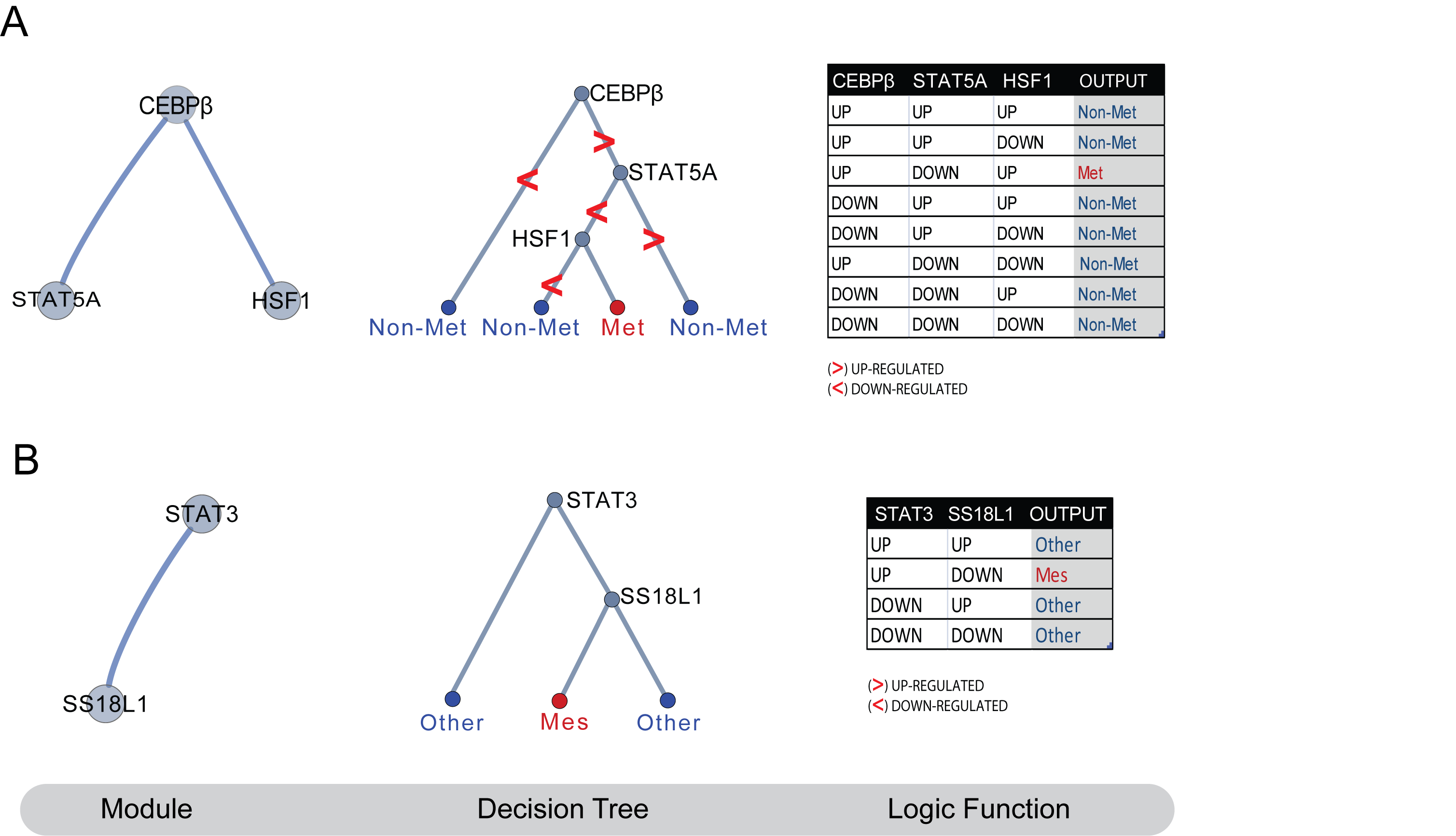

Supplement: Figure S1 — Modules, decision trees and logic functions. The logic functions behind key modules for breast cancer metastasis (A) or brain tumors (B) are represented using decision trees and truth tables. In each case the gene is interpreted as being up-regulated if its expression is above the threshold. Otherwise the gene is down-regulated. Each path from root to leaf in the tree maps to a different row in the truth table. Decision trees are typically not grown to the full extent and thus not all genes must be tested along each path if a subset of the genes is sufficient to determine the output. (TIF) [file pcbi.1002180.s001.tif]

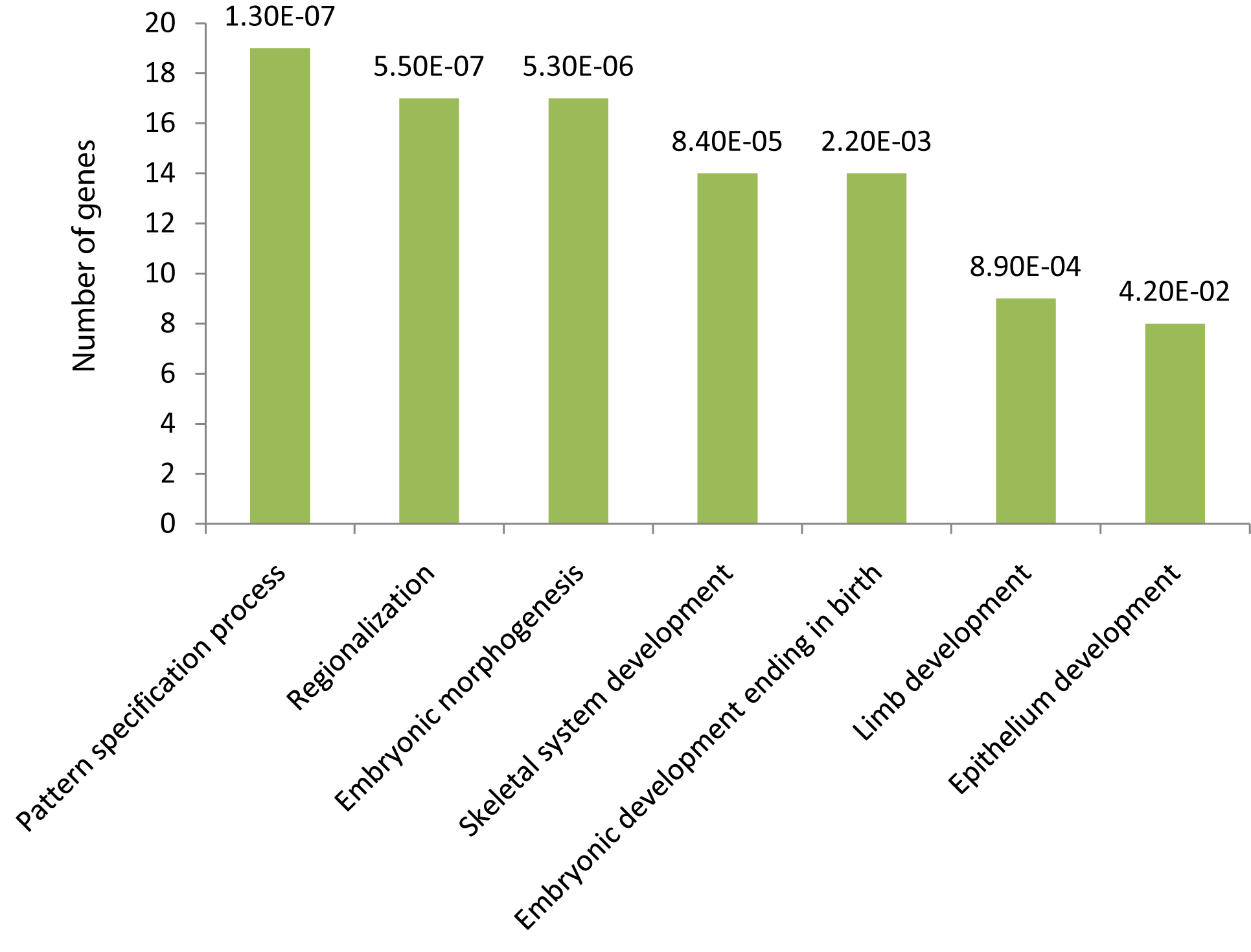

Supplement: Figure S2 — Gene Ontology enrichment analysis. Genes in the network identified by NGF ( Figure 3A ) are enriched for important developmental processes catalogued in the Gene Ontology. FDR is indicated above each bar. (TIF) [file pcbi.1002180.s002.tif]

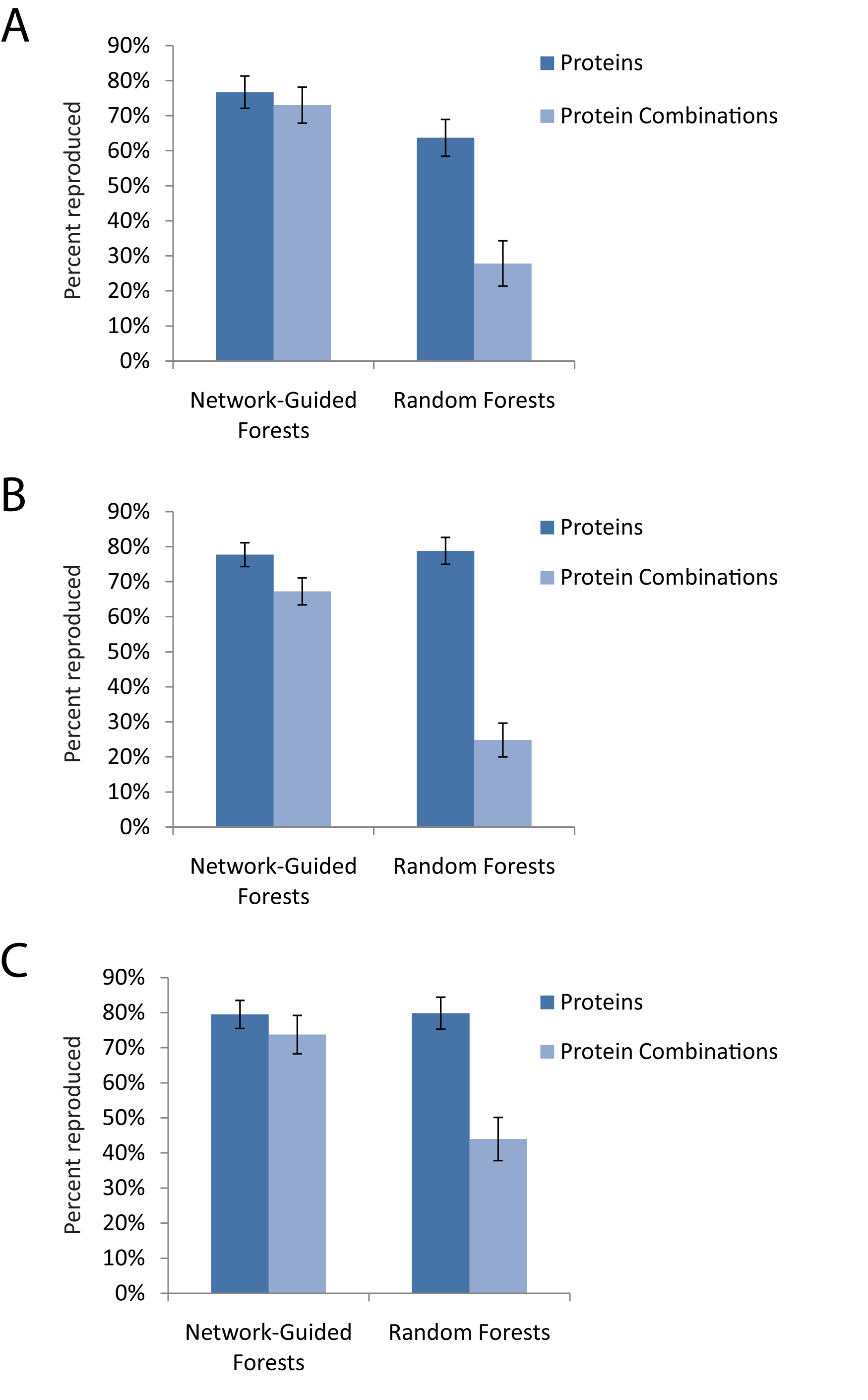

Supplement: Figure S3 — Robustness of NGF results in cross validation runs. The average percentage of the top 50 proteins and top 50 protein pairs identified for the developmental case study (A), the breast cancer metastasis case study (B) or the brain tumor case study (C) that were reproduced on datasets with 10% of the data held-out. Error bars indicate standard deviations estimated over 100 runs. (TIF) [file pcbi.1002180.s003.tif]

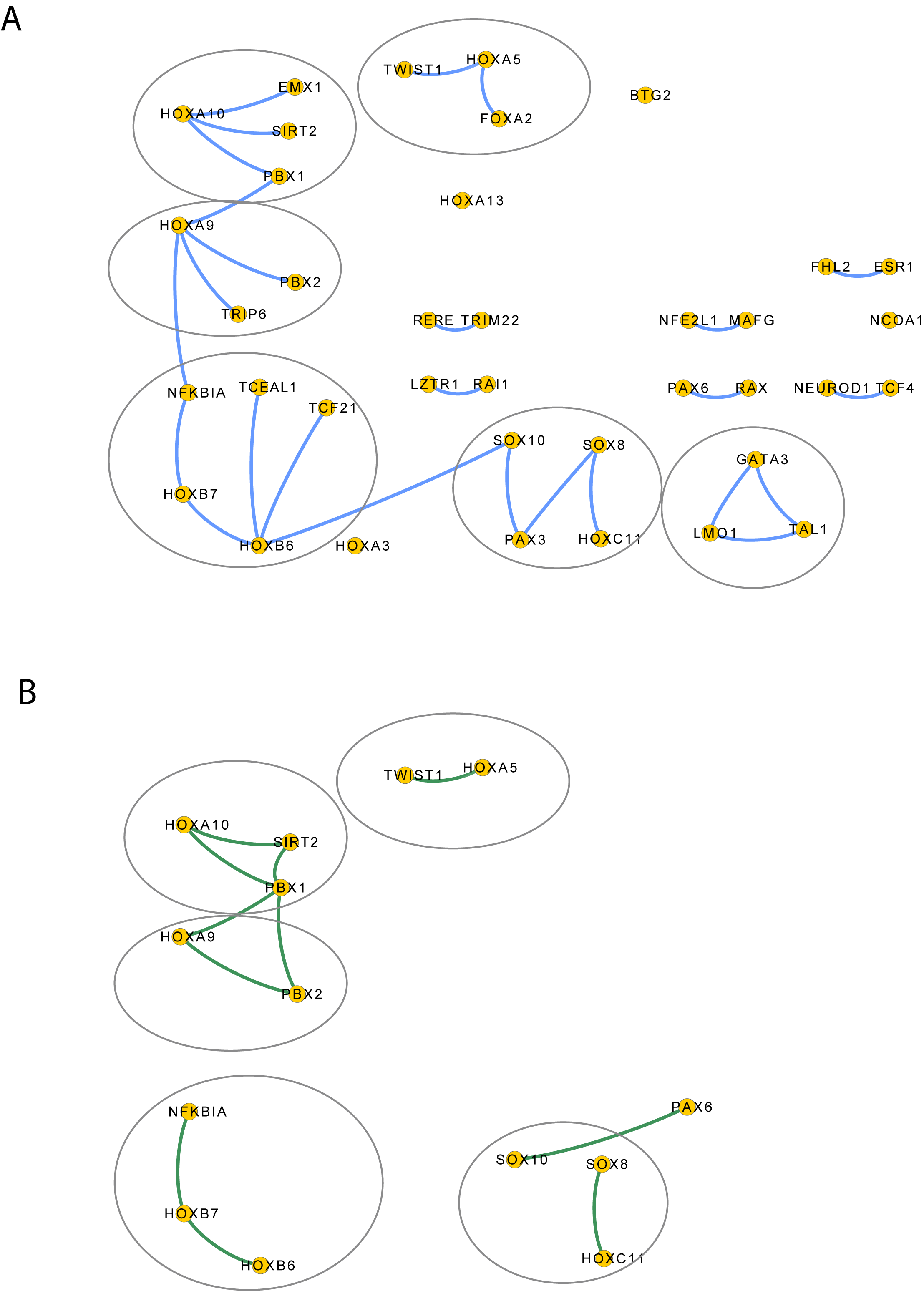

Supplement: Figure S4 — Overlap between NGF results based on Ravasi and Muller datasets. (A) Network modules identified using NGF based on the Ravasi dataset were limited to genes available also in the Muller dataset. Large modules (3 or more proteins) are encircled. (B) Overlapping genes and interactions identified based on the Muller dataset. Conserved large modules for which at least one interaction is retained in the result based on the Muller dataset are encircled. (TIF) [file pcbi.1002180.s004.tif]

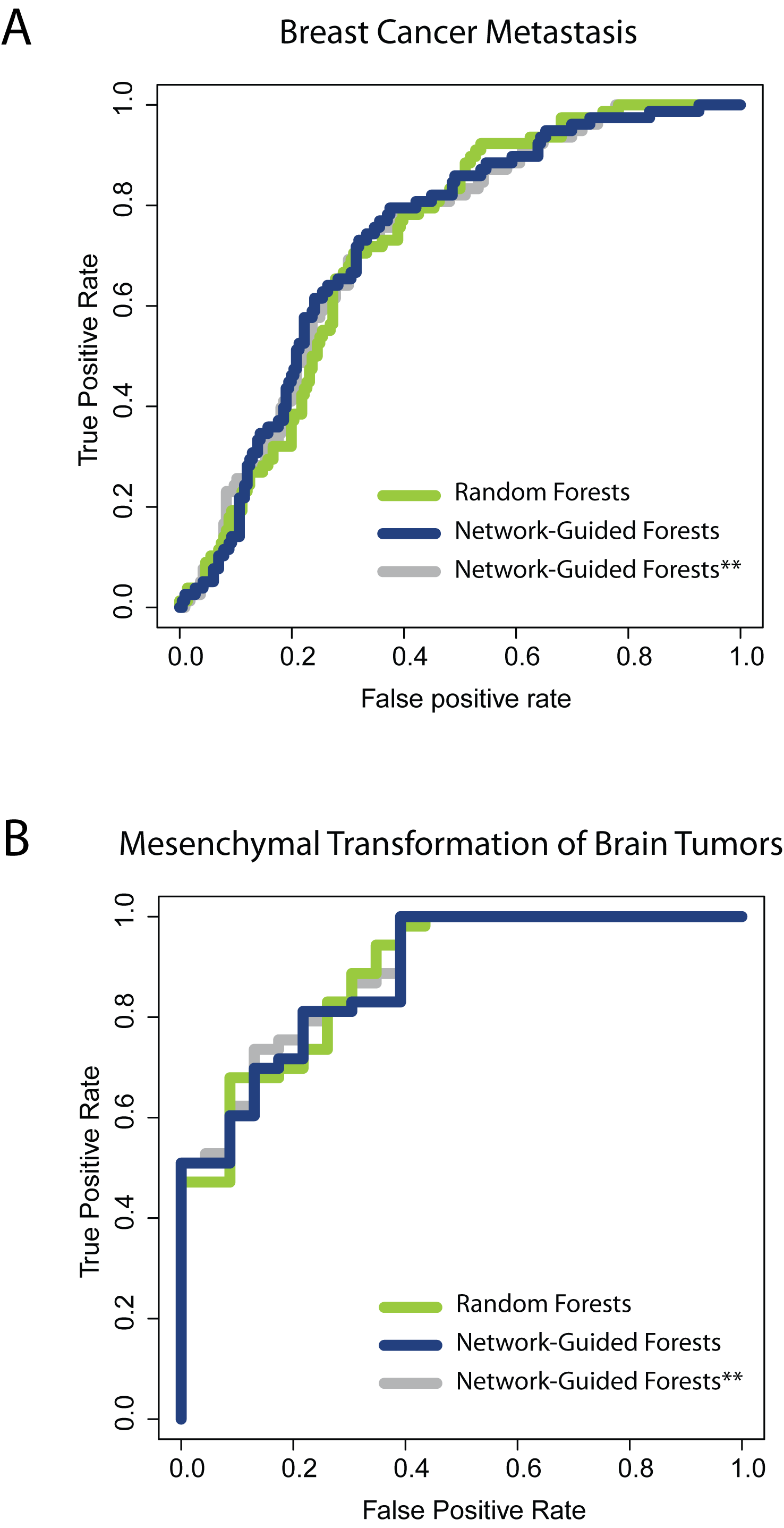

Supplement: Figure S5 — ROC analysis. Representative ROC curves for NGF, RF and NGF applied to networks with permuted edges (NGF**) for classification of breast cancer metastasis (A) and brain tumors (B). The average probability of a class computed across all trees in the forest is used as a parameter to trade off sensitivity and specificity. (TIF) [file pcbi.1002180.s005.tif]

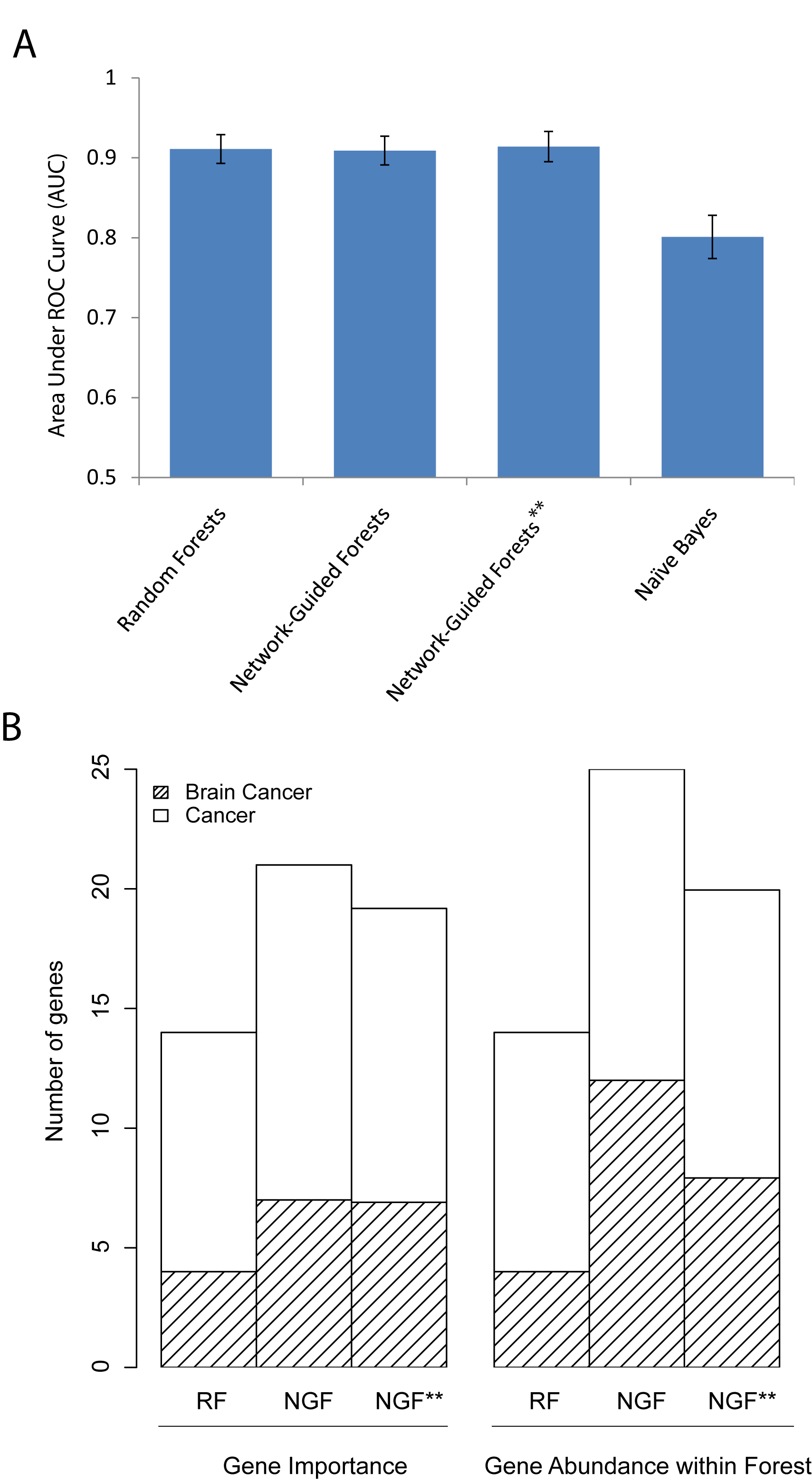

Supplement: Figure S6 — Classification performance and validation of network markers of mesenchymal transformation. (A) Average area under the ROC curve for NGF, RF, NGF applied to networks with permuted edges (NGF**), and Naïve Bayes (error bars denote standard deviation estimated over 100 runs). (B) Cancer and brain cancer associated genes identified among 100 top-scoring genes or 100 most abundant genes in the forest created using RF or NGF using the real network or networks with permuted edges (NGF**, average over 100 permutations is shown). (TIF) [file pcbi.1002180.s006.tif]

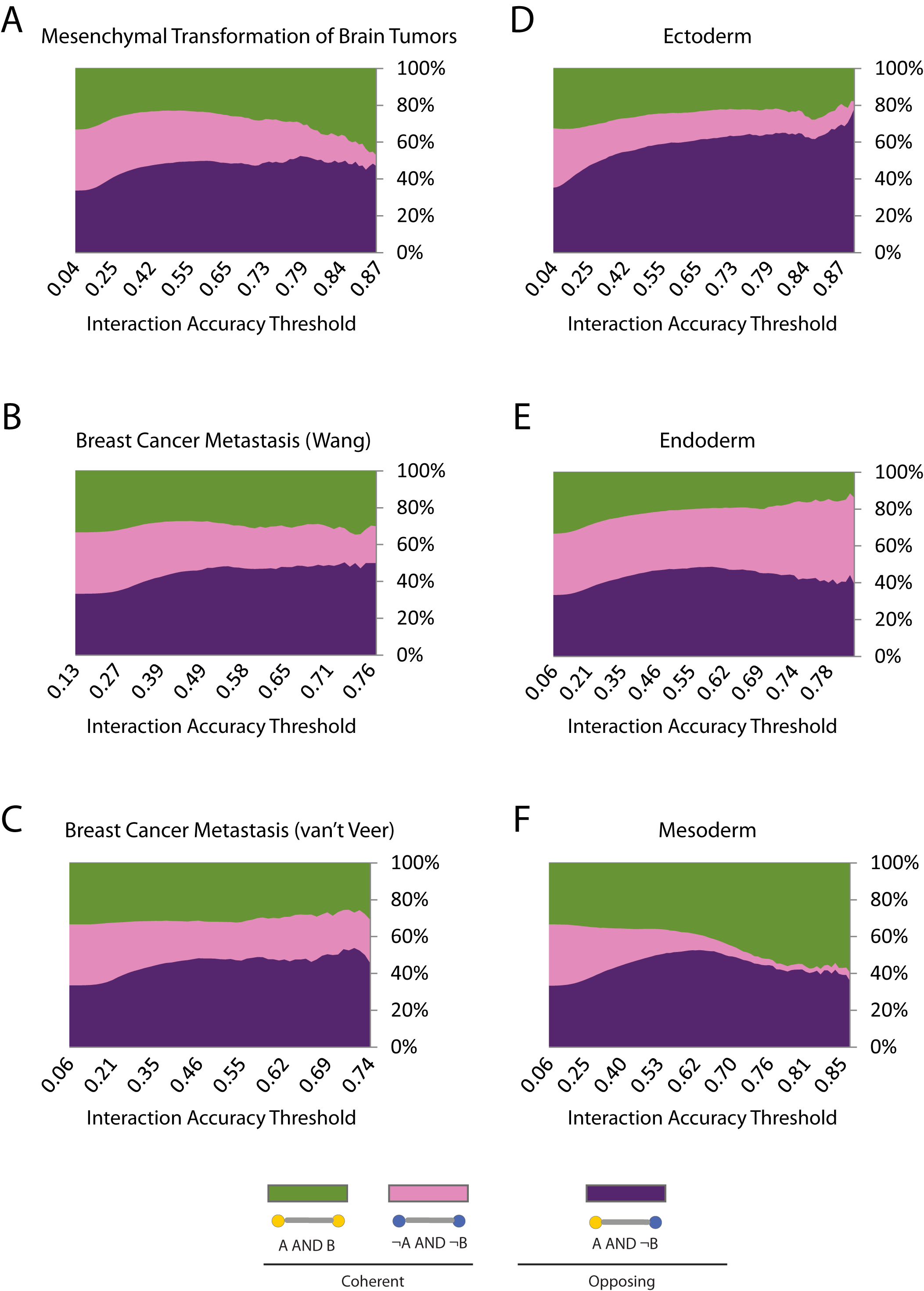

Supplement: Figure S7 — Network functions underlying development and cancer progression. For each study, the percentage of gene pairs assigned to each of the three functional categories is shown as a function of the score threshold used for selecting gene pairs. Accuracy is calculated as the average Laplace score over all trees in the forest (Text S1). (TIF) [file pcbi.1002180.s007.tif]
