# Supplementary material for: Protein Networks as Logic Functions in Development and Cancer
Source: PLoS Comput Biol. 2011 Sep 29;7(9):e1002180. doi: 10.1371/journal.pcbi.1002180 (PMC3182870; doi:10.1371/journal.pcbi.1002180)
Supplement: Table S1 — Protein-protein interaction networks and transcriptional profiles used in this study. (DOC) [file pcbi.1002180.s008.doc]

Table S1

| Study (Figures) | Protein-Protein Network | Transcriptional Profiles |
| --- | --- | --- |
| Development of germ layers (**Figures 2, 3, S2, S3, S4**) | (Ravasi et al. 2010)  1321 transcription factors  5227 TF-TF interactions | (Ravasi et al. 2010)  34 samples  1321 transcription factors  (Muller et al. 2008)  153 samples  7995 genes |
| Cancer progression (**Figures 4**, **S5, S6**) | (Chuang et al. 2007)  11,203 proteins  57,228 protein-protein interactions | (van 't Veer et al. 2002) *  295 samples  8141 genes  (Wang et al. 2005) *  286 samples  8141 genes  (Phillips et al. 2006)  76 samples  13,787 genes |
| Logic functions embedded in protein networks (**Figures 2, 5, S1, S7**) | (Ravasi et al. 2010) | (Ravasi et al. 2010)  (van 't Veer et al. 2002)  (Wang et al. 2005)  (Phillips et al. 2006) |

(*) indicates that the dataset was obtained and processed as in (Chuang et al. 2007).

**References**

Chuang HY, Lee E, Liu YT, Lee D, Ideker T (2007) Network-based classification of breast cancer metastasis. Mol Syst Biol 3: 140.

Muller FJ, Laurent LC, Kostka D, Ulitsky I, Williams R et al. (2008) Regulatory networks define phenotypic classes of human stem cell lines. Nature 455(7211): 401-405.

Phillips HS, Kharbanda S, Chen R, Forrest WF, Soriano RH et al. (2006) Molecular subclasses of high-grade glioma predict prognosis, delineate a pattern of disease progression, and resemble stages in neurogenesis. Cancer Cell 9(3): 157-173.

Ravasi T, Suzuki H, Cannistraci CV, Katayama S, Bajic VB et al. (2010) An atlas of combinatorial transcriptional regulation in mouse and man. Cell 140(5): 744-752.

van 't Veer LJ, Dai H, van de Vijver MJ, He YD, Hart AA et al. (2002) Gene expression profiling predicts clinical outcome of breast cancer. Nature 415(6871): 530-536.

Wang Y, Klijn JG, Zhang Y, Sieuwerts AM, Look MP et al. (2005) Gene-expression profiles to predict distant metastasis of lymph-node-negative primary breast cancer. Lancet 365(9460): 671-679.
