# Supplementary material for: Protein Networks as Logic Functions in Development and Cancer
Source: PLoS Comput Biol. 2011 Sep 29;7(9):e1002180. doi: 10.1371/journal.pcbi.1002180 (PMC3182870; doi:10.1371/journal.pcbi.1002180)
Supplement: Text S1 — Supplementary methods. (DOC) [file pcbi.1002180.s010.doc]

**Text S1**

**Analysis of germ layer-specific genes**

To assess how well NGF can identify genes that drive the development of each germ layer, we applied NGF three times, each time to score genes and gene pairs whose expression distinguishes one of the major layers (ectoderm, mesoderm or endoderm) from the others (case vs. control analysis). Next we analyzed 100 top-scoring genes and gene pairs and filtered them for genes which whose mean expression was higher in the cases than in controls. Functional enrichment analysis of these genes was performed using DAVID (Huang da et al. 2009) (**Figure 3C**).

**Reproducibility of results obtained for the developmental case study**

We first compared the reproducibility of results obtained using NGF to those based on standard Random Forests. To this end we checked the overlap between the lists of 50 top scoring single genes and gene pairs. We found that the network information did not affect the reproducibility of single top-scoring genes. However, it helped NGF identify 24% of the same gene pairs for both datasets, while standard Random Forests identified non-overlapping sets of top-scoring pairs. We further wanted to test whether the network constraint is overly-stringent, i.e., whether NGF is forced to identify the same gene pairs regardless of the expression data. To this end, we permuted the assignment of expression profiles to network nodes independently in each dataset, causing different nodes to become more informative. The resulting overlap between permuted and real data, for both single genes and pairs of genes, was insignificant. Thus, both the gene expression information as well as the protein-protein interaction network is needed for NGF to identify reproducible results.

**Analysis of functional gene combinations**

To identify functional gene combinations that were predictive of class, we analyzed pairs of genes of the type (A, B) where one gene was selected as the root node in a forest decision tree and the other was its direct descendant. These gene pairs were categorized according to sign of gene expression as determined by the decision tree. The three functional combinations were: “A AND B”, “NOT A AND NOT B”, “A AND NOT B”. Each functional combination determined a decision rule that could be used to classify samples. For instance “A AND NOT B” indentifies samples in which the expression of gene A is above the threshold and the expression of gene B is below the threshold. We asked which of these decision rules could best separate the samples into class-homogeneous groups and which types of decision were preferred in each biological case study. To measure the accuracy of each decision rule with respect to class ***c***, we applied the Laplace accuracy measure [e.g. (Clarke and Boswell 1991)]:

*L_c_  = (n_c_ + 1) / (N + k),*

Where *N* is the total number of samples covered by the rule, *n_c_* is the number of covered samples that are in the class *c,* and *k* is the total number of classes (in our case *k* = 2). This score is averaged over all trees in which a given rule is present.

# References

# Clarke P, Boswell R. Rule Induction with CN2: Some Recent Improvements. In: Kodratoff Y, editor; 1991. Springer-Verlag.

# Huang da W, Sherman BT, Lempicki RA (2009) Systematic and integrative analysis of large gene lists using DAVID bioinformatics resources. Nat Protoc 4(1): 44-57.
